# Prevalence of Metabolic Syndrome and Its Associated Factors Among Malagasy Patients With Ischemic Stroke: A Cross-Sectional Study

**DOI:** 10.7759/cureus.90462

**Published:** 2025-08-19

**Authors:** Sitraka Angelo Raharinavalona, Rija Eric Raherison, Thierry Razanamparany, Zo Andrianina Rahobiarivony, Dave Patrick Rakotomalala

**Affiliations:** 1 Endocrinology Department, Soavinandriana Hospital Center, Antananarivo, MDG; 2 Endocrinology Department, Joseph Raseta Befelatanana University Hospital Center, Antananarivo, MDG

**Keywords:** associated factors, cardiovascular risk factors, ischemic stroke, madagascar, metabolic syndrome

## Abstract

Introduction

Metabolic syndrome (MetS) and ischemic stroke are significant public health issues due to their high prevalence and impact on morbidity and mortality. The objective of this study was to determine the prevalence of MetS and its associated factors among Malagasy patients with ischemic stroke.

Materials and methods

This cross-sectional study was conducted over a three-year period at the Soavinandriana Hospital Center, Antananarivo, Madagascar. MetS was defined according to the International Diabetes Federation’s 2009 criteria. Ischemic stroke was confirmed by brain computerized tomography. The TOAST classification was used to determine the etiologies.

Results

We included 307 patients with ischemic stroke. The prevalence of MetS was 77.9%. According to the logistic regression analysis, the factors associated with MetS were female gender (adjusted odds ratio (aOR) = 3.99, p-value = 0.0031), hypertension (aOR = 4.02, p-value = 0.0029), overweight or obesity (aOR = 2.13, p-value = 0.0122), diabetes (aOR = 4.20, p-value = 0.0011), Glasgow scale ≤10 (aOR = 2.18, p-value = 0.0157), National Institutes of Health Stroke Scale (NIHSS) ≥15 (aOR = 2.94, p-value = 0.0086), and modified Rankin scale ≥ 3 (aOR = 2.29, p-value = 0.0033). As components of MetS, elevated blood pressure was significantly associated with overweight or obesity (aOR = 1.97, p-value = 0.0440), large artery atherosclerosis (LAA) (aOR = 10.2, p-value = 0.0256), and cardioembolism (aOR = 0.25, p-value = 0.0177). The associations between elevated fasting glucose and LAA (aOR = 2.26, p-value = 0.0012) and cardioembolism (aOR = 0.34, p-value = 0.0004). Elevated triglycerides was associated significantly with diabetes (aOR = 2.01, p-value = 0.0452), cardioembolism (aOR = 0.45, p-value = 0.0115), and lacunar infarcts (aOR = 3.05, p-value = 0.0336).

Conclusion

Screening for MetS should be a routine part of assessing patients with ischemic stroke. Proper management of MetS and its components could lead to improvements in the profile and prognosis of ischemic stroke.

## Introduction

Metabolic syndrome (MetS) combines morphological, physiological, and biochemical abnormalities [1]. Each component of MetS is an independent risk factor for cardiovascular diseases, and when combined, they increase the rates and severity of cardiovascular diseases [2]. MetS prevalence depends on age, gender, ethnicity, and diagnostic criteria. In the general population, around a third of adults suffer from MetS [3]. In Africa, this prevalence fluctuated between 6.0% and 36.5% [4,5]. In Madagascar, a study of type 2 diabetics showed a higher prevalence of MetS at 86.30% [6].

MetS has become increasingly prevalent as people's lives improve and is associated with an increased risk of all-cause mortality [7]. It activates the sympathetic nervous system and the renin-angiotensin system, while also increasing levels of adipokines and pro-inflammatory cytokines [8]. In addition, ischemic stroke and MetS share a common molecular profile and genetic association [9]. Therefore, MetS predisposes individuals to atherosclerosis and its complications [2,8]. MetS is significantly associated with the occurrence of carotid atherosclerosis, which is more severe [10]. It is also a factor in the occurrence, poor prognosis, and recurrence of ischemic stroke [11].

In sub-Saharan Africa, as in Madagascar, there is a lack of references on this topic. Stroke is one of the most common causes of mortality in hospitals. Indeed, their management is very limited. Almost all ischemic strokes are delayed. Cerebral magnetic resonance imaging (MRI) and thrombolysis are unavailable to the entire Malagasy population. The purpose of this study is to determine the prevalence of MetS and its associated factors among Malagasy patients with ischemic stroke.

## Materials and methods

Study design and setting

A cross‐sectional analytical study was conducted at the Cardiovascular Diseases and Internal Medicine departments of the Soavinandriana Hospital Center (Military Hospital) in Antananarivo, Madagascar. These departments are considered references for managing cardiovascular, internal medicine, metabolic, and endocrine diseases in the capital city and throughout the country of Madagascar. The study was carried out for three years, from January 2021 to December 2023.

Study population

Our study population consists of patients suffering from an ischemic stroke who were tested for MetS. The diagnosis of ischemic stroke was confirmed in patients with sudden-onset focal neurological signs such as motor and/or sensory deficits and by parenchymal hypodensity corresponding to one or more affected arterial territories on brain computerized tomography (CT) [12]. MetS is defined according to the IDF 2009 criteria, which include the presence of at least three of the following criteria: waist circumference (WC) ≥ 94 cm in men / ≥ 80 cm in women; triglycerides ≥ 150 mg/dL (1.7 mmol/L) and/or drug treatment for elevated triglycerides; high-density lipoprotein cholesterol (HDL-C) < 40 mg/dL (1.0 mmol/L) in men, < 50 mg/dL (1,29 mmol/L) women and/or drug treatment for reduced HDL-C; blood pressure (BP) ≥ 130/85 mmHg and/or antihypertensive drug treatment in a patient with a history of hypertension; fasting glucose (FG) ≥ 100 mg/dL (5.5 mmol/L) and/or drug treatment of elevated glucose [13]. Patients with edema, pregnancy, hypothyroidism, any malignancy that might influence lipid parameters, or incomplete records were excluded from the study.

The population sampling was exhaustive. The sample size was determined using a single population proportion formula: $n=(Z1-\alpha/2)^{2} P(1-P)/d^{2}$. This was based on the assumptions of a 95% confidence level, $(Z1-\alpha/2)^{2}$ = 1.96, a 5% margin of error, and a 27.7% prevalence of MetS [14]. The final sample size for the study was 307 patients who consented to participate.

Clinical and laboratory data

The variables studied included demographic data (age, gender), cardiovascular risk factors (hypertension, diabetes mellitus, dyslipidemia, smoking, overweight/obesity, menopause, microalbuminuria), MetS, and ischemic stroke characteristics.

The waist circumference was measured using a tape measure with constant tension while the patient was standing, if possible, at a point halfway between the lower base of the last rib and the iliac crest. The body mass index (BMI) was calculated by dividing the subject's weight in kilograms by their height in meters squared. Overweight and obesity were defined as BMI values ranging from 25 to 29.9 kg/m² and greater than or equal to 30 kg/m², respectively. Blood pressure was measured by an investigator in a patient who remained silent during and between measurements, in a calm, semi-sitting position for a period of between three and five minutes, in a comfortable room temperature environment, after the patient had rested and not eaten for 30 minutes and after urinating. The measurements were obtained using an Omron® electronic blood pressure monitor, equipped with a suitable upper arm cuff.

The presence of hypertension was confirmed by a blood pressure reading of ≥140/90 mmHg (based on an average of ≥2 measurements obtained on ≥2 occasions) or by the use of antihypertensive medication. Fasting glucose and glycated hemoglobin (HbA1c) levels were measured using the colorimetric technique on Abbott Alinity C 8501 and high-performance liquid chromatography (HPLC), respectively. The diagnosis of diabetes was determined by the patient’s self-report using antidiabetic medication or based on the diagnostic criteria of the American Diabetes Association [15]. Lipid parameters were assessed using the colorimetric technique on Abbott Alinity C 8502. Patients with low-density lipoprotein cholesterol (LDLc) levels outside the targets recommended by the European Society of Cardiology [16] or those taking lipid-lowering medication were considered to have dyslipidemia. Body mass index (BMI) was calculated as weight in kilograms (kg) divided by the square of height in meters (m²). National Institutes of Health Stroke Scale (NIHSS) and modified Rankin scale were used for the assessment. The Trial of Org 10172 in Acute Stroke Treatment (TOAST) classification was utilized for etiologies categorization of ischemic stroke: large artery atherosclerosis, cardioembolism, lacunar infarcts, others, undetermined [17].

Statistical analysis

Data were collected from patient medical records using a pre-established survey form and analyzed using Epi Info™ version 3.5.4 software (United States Centers for Disease Control and Prevention in Atlanta, Georgia). Continuous variables were presented as median with interquartile range (IQR) 25% and 75%. Categorical variables were expressed as frequencies and percentages. To determine the impact of MetS on ischemic stroke and the association between MetS and carotid atherosclerosis, ANOVA, chi-square/Fisher’s exact, and t-tests were used with a significance threshold of less than 0.05. Crude (cOR) and adjusted odds ratios (aOR) with their respective 95% confidence intervals (CI) were calculated. Candidate risk factors for multivariate analysis were identified in bivariate analysis.

Ethical considerations

The study adhered to the principles outlined in the Declaration of Helsinki. Prior to conducting the study, a request for authorization to collect data was submitted and approved by the General Director of the hospital and the head of the department. The patients were informed about the purpose of the study. Patients’ anonymity and confidentiality were maintained, and the Review Board of Soavinandriana Hospital also approved the study (No: 098/CENHOSOA/DG/DT on December 23, 2022). Consent was obtained from all participants.

## Results

**Figure 1:**
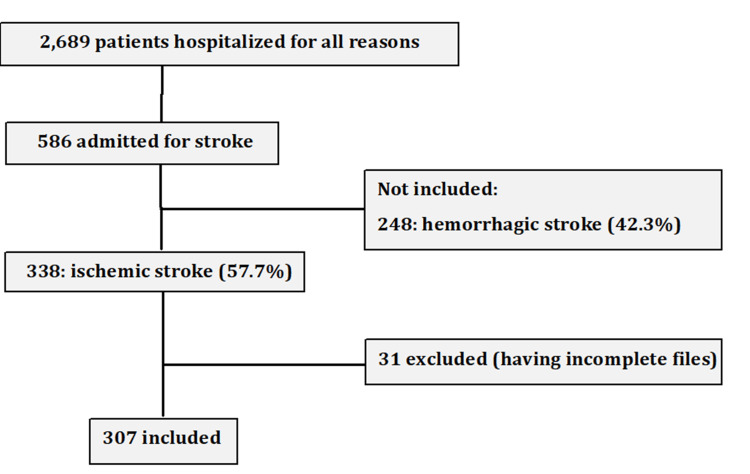
Flowchart for the study participants

During the study period, 338 patients were admitted for an ischemic stroke, of whom 307 (90.8%) were included and 31 (9.2%) were excluded (see Figure 1).

**Figure 2:**
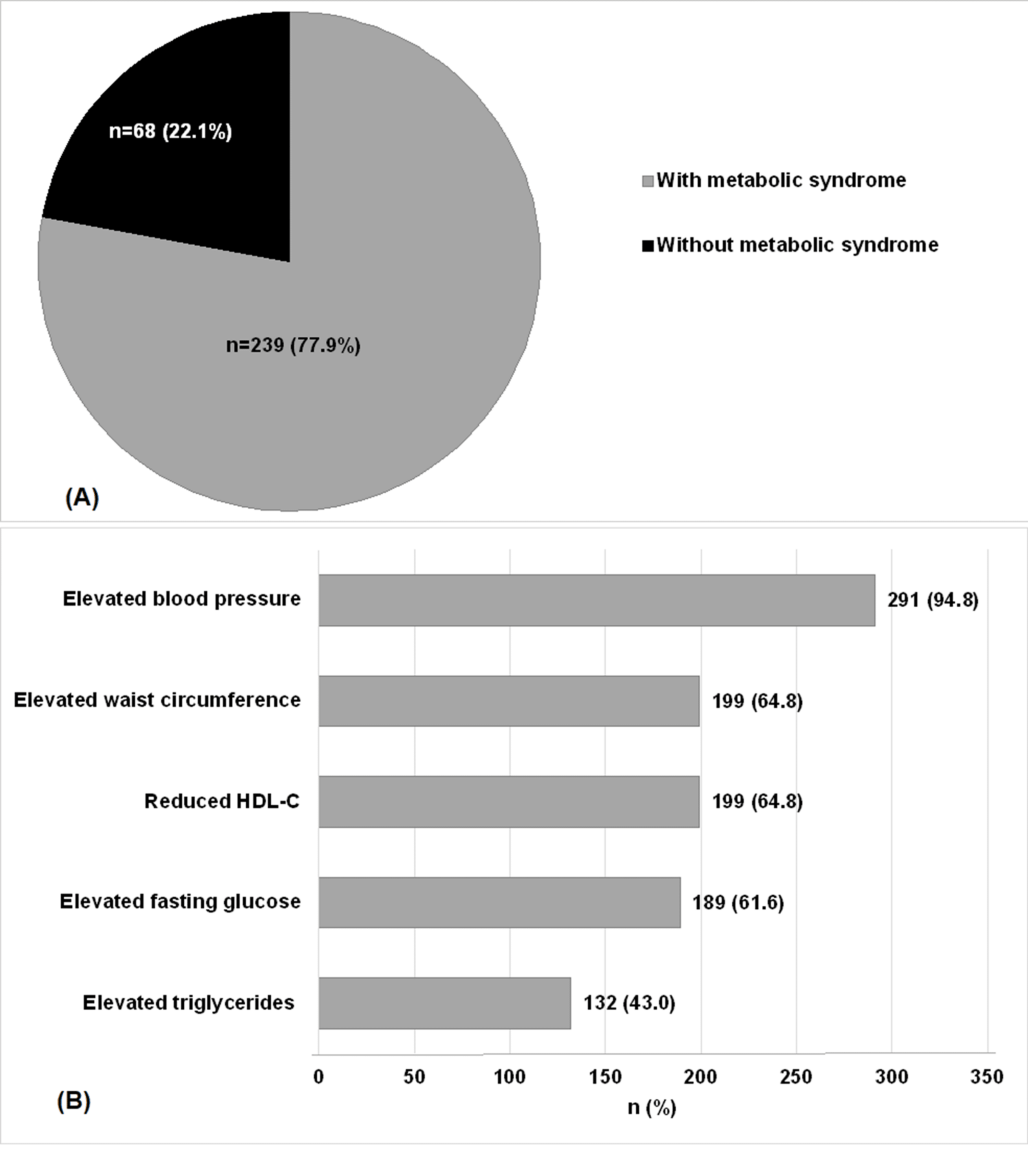
Metabolic syndrome: (A) prevalence, (B) components

Two hundred and thirty-nine patients (77.9%) had MetS. The most frequent component of MetS was elevated blood pressure, present in 94.8% of cases (see Figure 2).

The majority of the patients were male (65.5%), with a median age of 65. Patients with MetS had significantly worse Glasgow, NIHSS, and modified Rankin scores. In the overall population studied, large artery atherosclerosis was the most frequent etiology of ischemic stroke in 40.1% of cases, followed by cardioembolism in 20.2%. None of the patients received thrombolysis, as it was not available at the study site. The in-hospital mortality rate was 7.8%. Table 1 shows the general characteristics of the study population.

**Table 1:** General characteristics of the study population ^*^significant p-value <0.05. p-values were calculated using the chi-square test. IQR = interquartile range, MetS = metabolic syndrome, NIHSS = National Institutes of Health Stroke Scale, RAS = renin-angiotensin system

Variables	Total N = 307	With MetS n = 239	Without MetS n = 68	Chi-square	p-value
Female gender, n (%)	106 (34.5)	96 (40.2)	10 (14.7)		0.0011*
Age ≥60 years, n (%)	206 (67.1)	162 (67,8)	44 (64.7)	0.109	0.3675
Cardiovascular risk factors					
Hypertension, n (%)	285 (92.8)	228 (95.4)	57 (83.8)	8.991	0.0016*
Dyslipidemia, n (%)	166 (54.1)	126 (52.7)	40 (58.8)	0.567	0.2259
Smoking, n (%)	105 (34.2)	75 (31.4)	30 (44.1)	3.271	0.0364*
Menopause, n (%)	89 (29.0)	74 (31.0)	15 (22.1)	1.629	0.0993
Overweight or obesity, n (%)	88 (28.7)	75 (31.4)	13 (19.1)	3.317	0.0317*
Diabetes, n (%)	81 (26.4)	74 (31.0)	7 (10.3)	10.61	0.0001*
Scales					
Glasgow ≤10, n (%)	81 (26.41)	71 (29.7)	10 (14.7)	5.385	0.0055*
NIHSS ≥15, n (%)	89 (29.0)	81 (33.9)	8 (11.8)	11.53	0.0001*
Modified Rankin ≥ 3, n (%)	105 (34.2)	93 (38.9)	12 (17.6)	9.713	0.0004*
Etiologies of ischemic stroke					
Large artery atherosclerosis, n (%)	123 (40.1)	96 (40.2)	27 (39.7)	0.005	0.5306
Cardioembolism, n (%)	62 (20.2)	43 (18.0)	19 (28.0)	2.663	0.0541
Lacunar infarcts, n (%)	18 (5.9)	16 (6.7)	2 (2.9)	0.756	0.1958
Undetermined, n (%)	104 (33.8)	84 (35.1)	20 (29.4)	0.467	0.2483
Treatment					
RAS blockers, n (%)	250 (81.4)	198 (82.8)	52 (76.5)	1.032	0.1547
Calcium channel blockers, n (%)	202 (65.8)	161 (67.4)	41 (60.3)	0.882	0.1734
Beta blocker, n (%)	116 (37.8)	93 (38.9)	23 (33.8)	0.386	0.2684
Statins, n (%)	254 (82.7)	202 (84.5)	52 (76.5)	1.871	0.0882
Antiplatelet agents, n (%)	254 (82.7)	201 (84.1)	53 (77.9)	1.008	0.1575
In-hospital outcomes					
Mortality, n (%)	24 (7.8)	21 (8.8)	3 (4.4)	0.864	0.1776

According to the logistic regression analysis, the factors associated with MetS were female gender (aOR = 3.99, p-value = 0.0031), hypertension (aOR = 4.02, p-value = 0.0029), overweight or obesity (aOR = 2.13, p-value = 0.0122), and diabetes (aOR = 4.20, p-value = 0.0011). However, large artery atherosclerosis (cOR = 1.02, p-value = 0.5306, cardioembolism (cOR = 0.57, p-value = 0.0541), lacunar infarcts (cOR = 2.36, p-value = 0.1958), and in-hospital mortality (cOR = 2.08, p-value = 0.1776) were not influenced by MetS (see Table 2).

**Table 2:** Factors associated with metabolic syndrome *significant p-value <0.05. p-values were calculated using the logistic regression analysis. aOR = adjusted odds ratio, CI = confidence Interval, NIHSS = National Institutes of Health Stroke Scale, OR = odds ratio

Variables	Bivariate analysis		Multivariate analysis	
	OR (95% CI)	p value	aOR (95% CI)	p value
Female gender	3.96 (1.68-9.16)	0.0011^*^	3.99 (1.58-9.77)	0.0031^*^
Age ≥60 years	1.15 (0.62-2.09)	0.3675		
Hypertension	3.97 (1.48-10.6)	0.0016^*^	4.02 (1.61-10.1)	0.0029^*^
Dyslipidemia	0.78 (0.43-1.39)	0.2259		
Smoking	0.58 (0.32-1.11)	0.0364^*^	0.61 (0.34-1.09)	0.0983
Menopause	1.58 (0.81-3.22)	0.0993		
Overweight or obesity	1.93 (1.01-3.87)	0.0317^*^	2.13 (1.03-5.55)	0.0122^*^
Diabetes	3.89 (1.67-10.6)	0.0001^*^	4.20 (1.77-9.92)	0.0011^*^
Glasgow scale ≤10	2.44 (1.15-5.67)	0.0055^*^	2.18 (1.04-4.01)	0.0157^*^
NIHSS ≥15	3.83 (1.71-9.73)	0.0001^*^	2.94 (1.08-9.99)	0.0086^*^
Modified Rankin scale ≥ 3	2.96 (1.47-6.41)	0.0004^*^	2.29 (1.06-9.98)	0.0141^*^
Large artery atherosclerosis	1.02 (0.57-1.84)	0.5306		
Cardioembolism	0.57 (0.29-1.12)	0.0541		
Lacunar infarcts	2.36 (0.53-21.7)	0.1958		
Mortality	2.08 (0.59-11.2)	0.1776		

**Figure 3:**
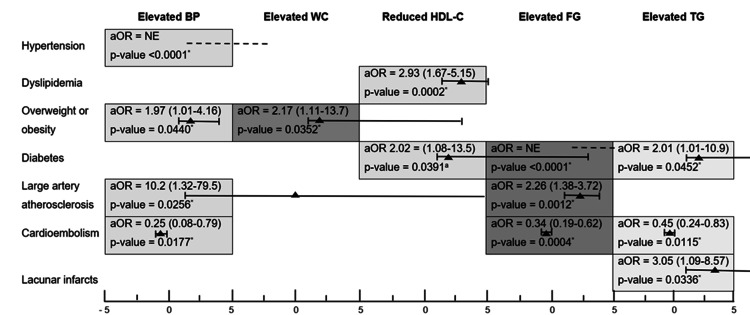
Factors associated with components of metabolic syndrome. *significant p-value <0.05. p-values were calculated using the logistic regression analysis. aOR = adjusted odds ratio, BP = blood pressure, CI = confidence Interval, FG = fasting glucose, HDL-C = high-density lipoprotein cholesterol, NE = not estimable, TG = triglycerides, WC = waist circumference

Large artery atherosclerosis was significantly associated with elevated BP (aOR 10.2, p-value = 0.0256) and elevated FG (aOR 2.26, p-value = 0.0102). Elevated BP (aOR 0.25, p-value = 0.0177), elevated FG (aOR 0.34, p-value = 0.0004), and elevated triglycerides (aOR 0.45, p-value = 0.0115) were protective factors for cardioembolism. Figure 3 summarizes the factors associated with components of MetS.

## Discussion

The prevalence of MetS found in the present study was somewhat similar to those of studies carried out in Pakistan (66.96%) and Iran (62%) [18,19] However, it was less frequent in populations with stroke, such as in Japan (45.5%) [20] and China (31.4%) [21]. Elevated BP remains the most frequent component of MetS in this and other studies [22,23]. This was not found by other authors [18,19]. This disparity could probably be linked to differences in the populations studied, their genetic susceptibilities to developing the various MetS components, the study periods, the study settings, and, above all, the MetS criteria used.

In the present study, female gender, hypertension, overweight or obesity, and diabetes were found to be 3.99, 4.02, 1.89, and 3.92 times more prevalent in patients with MetS. This correlation has also been noted by other researchers, who found that female gender and older age were associated with a higher likelihood of developing MetS [22]. Lim and Cheah reported that the risk of MetS increased by 3% for each year of age [24]. The balance between testosterone and estrogen levels plays a significant role in the distribution of adipose tissue and lipid levels in the body. Women tend to accumulate subcutaneous adipose tissue with age, regardless of their menopausal status, while visceral adipose tissue tends to increase after menopause, coinciding with a decrease in 17-estradiol levels [25]. The presence of hypertension and diabetes as components of MetS further supports their significant associations with the condition. In a study conducted in Seoul, Korea, it was found that an increase in body mass index was associated with a 2.84-fold increase in the risk of developing MetS [26].

The various clinical stroke scales were significantly worse in patients with MetS in this and other studies [27,28]. Indeed, MetS increases the risk of cerebrovascular and cardiovascular disease severity. In the present study, MetS itself did not influence ischemic stroke etiologies, but elevated BP and elevated FG were risk factors for large artery atherosclerosis and protective factors for cardioembolism. Elevated triglycerides were significantly associated with lacunar infarcts. In the study by Maruyama et al., hypertension and fasting hyperglycemia tripled the risk of acute ischemic noncardioembolic stroke [20]. He et al. also reported significant associations between elevated WC, elevated BP, and elevated triglycerides and large artery stroke, between elevated BP and cardioembolic stroke, and between elevated BP, elevated triglycerides, and small vessel stroke [29]. Furthermore, a high triglyceride and glucose index, as a biochemical marker of insulin resistance, is associated with an increased risk of ischemic stroke in the general population [30]. In both the present study and that of Mi et al. [22], the association between MetS and mortality after ischemic stroke was not significant. The absence of a significant association between MetS and in-hospital mortality in the present study could be explained by the size of the sample.

The present study was one of the first to be conducted on this subject in Madagascar. This has helped to streamline the country's databases and enhance patient care. This work has inspired us to initiate other studies in our country, particularly on a national scale, with larger samples and more sophisticated methods.

Despite its contributions, the present study was conducted in a tertiary hospital, which may limit the generalizability of the results to the entire population. Selection bias may also occur. The cross-sectional observational design of the study precludes the establishment of causal relationships between MetS and ischemic stroke. The absence of a control group for comparison with stroke-free individuals limited the study. The lack of thrombolysis availability at the study site may relate to the clinical presentation of stroke and patient outcomes. It is evident that certain potential factors, including dietary habits, physical activity, and socioeconomic status, were not included in the patient records.

## Conclusions

This study highlighted a high prevalence of MetS in patients with ischemic stroke and significant associations between MetS, gender, cardiovascular risk factors, and poor clinical outcomes. In addition, these components were associated with the etiology of ischemic stroke. Consequently, systematic screening of subjects for MetS and its components is particularly important in female subjects with classic cardiovascular risk factors. The effective management of MetS and its components is imperative to improve the functional and vital prognosis of patients with ischemic stroke, requiring a multidisciplinary approach.
